# Host-adaptive mutations in Chikungunya virus genome

**DOI:** 10.1080/21505594.2024.2401985

**Published:** 2024-09-12

**Authors:** Xinhang Ning, Binghui Xia, Jiaqi Wang, Rong Gao, Hao Ren

**Affiliations:** a Department of Microbiology, Faculty of Naval Medicine, Shanghai Key Laboratory of Medical Biodefense, Naval Medical University, Shanghai, People’s Republic of China; b Department of Respiratory Medicine, The People’s Liberation Army Joint Logistic Support Force 943 Hospital, Wuwei, Gansu, People’s Republic of China

**Keywords:** Chikungunya virus, arbovirus, adaptive mutation, evolution

## Abstract

Chikungunya virus (CHIKV) is the causative agent of chikungunya fever (CHIKF), and its primary vectors are the mosquitoes Aedes aegypti and Aedes albopictus. CHIKV was initially endemic to Africa but has spread globally in recent years and affected millions of people. According to a risk assessment by the World Health Organization, CHIKV has the potential seriously impact public health. A growing body of research suggests that mutations in the CHIKV gene that enhance viral fitness in the host are contributing to the expansion of the global CHIKF epidemic. In this article, we review the host-adapted gene mutations in CHIKV under natural evolution and laboratory transmission conditions, which can help improve our understanding of the adaptive evolution of CHIKV and provide a basis for monitoring and early warning of future CHIKV outbreaks.

Chikungunya virus (CHIKV) is an enveloped, single-stranded RNA virus of the genus *Alphavirus* in the family Togaviridae and is transmitted through the bite of the mosquitoes *Aedes albopictus* or *Aedes aegypti* [1]. Infection in humans by CHIKV can lead to chikungunya fever (CHIKF), characterized primarily by fever, rashes, and joint pain [2]. Other symptoms include headache, myalgia, nausea and vomiting, conjunctivitis, and meningitis. Its impacts on the central nervous system are the most harmful [3], and severe acute cases may progress to heart and multi-organ failure [4].

CHIKV was first isolated from a febrile patient in Tanzania in 1952 and has since become endemic only in parts of Africa and Southeast Asia [5,6]. A CHIKF outbreak in eastern Kenya in 2004 was followed by a CHIKV pandemic in the region surrounding the Indian Ocean [7], and a large outbreak on the French island of Réunion in 2005–2006, affecting hundreds of thousands of people [8,9]. The first local transmission of CHIKV occurred in the United States in 2013 and subsequently spread to Central and South America, the Caribbean, and beyond [10]. CHIKF is currently endemic in various regions of Asia, Africa, Europe, and the Americas. According to the World Health Organization, CHIKV has been detected in approximately 60 countries worldwide since 2004, affecting millions of people [11].

Changing host adaptation is a strategy through which viruses expand their host range and endemic area [12]. Genetic mutation of several or even only a single amino acid is one of the primary ways by which the host range of a virus can be expanded or by which the virus undergoes changes in pathogenicity. For example, the alanine-to-valine mutation at residue 188 of a non-structural protein of the Zika virus over the course of its evolution enhanced its infection of mosquitoes and replication in mice and is a cause of the accelerated endemicity of the Zika virus [13]. The S139N mutation in the prM protein, which appeared during a Zika virus epidemic in the Americas, resulted in a significant increase in the viral infectivity of neural progenitor cells in mice and humans, more severe microcephaly in mouse foetuses, and increased mortality in neonatal mice [14]. Other arboviruses such as dengue virus also expand their infected population and endemic area through changes in host adaptation [15]. In addition to vector-borne viruses, other RNA viruses such as human immunodeficiency virus, hepatitis C virus, and poliovirus have developed changes in host adaptation [16–18].

## CHIKV genomic structure

**Figure 1.**
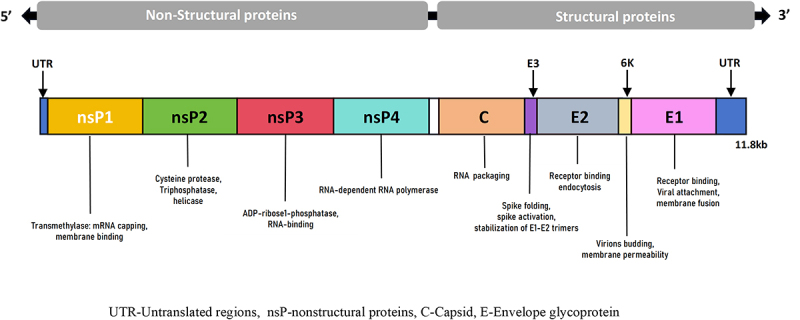
Genomic organization of CHIKV.

CHIKV is a single-stranded positive-sense RNA virus with a diameter of approximately 70 nm and a total genome length of approximately 11.8 kb, which includes untranslated regions (UTR) at the 5' and 3' ends and two open reading frames, ORF1 and ORF2, separated by a non-coding region [19,20]. Stem-loop RNAs formed from conserved sequence elements in the 5’ UTR, 3’ UTR, and sub-genomic promoters in the non-coding region, regulate replication and transcription of the viral genome. ORF1 encodes non-structural proteins (nsP1, nsP2, nsP3, and nsP4), and ORF2 encodes structural proteins, namely, capsid protein (C), envelope glycoproteins (E), and 6K/Transframe (TF) protein (Figure 1 [21]). The four non-structural proteins make up the replication complex. nsP1 has transmethylase activity and is the main enzyme involved in mRNA capping [22]; it also anchors the viral replication enzyme complex to the lipid membranes [23]. nsP2 has RNA helicase activity as well as cysteine protease and nucleotide triphosphatase activity, and can inhibit the immune response of the host cell. nsP3 plays a role in ADP ribose hydrolysis and RNA binding, while it promotes viral infection by interacting with some host cell proteins [24]. nsP4 has RNA polymerase activity and catalyzes RNA synthesis.These proteins assemble into viral replication complexes to synthesize full-length negative-strand RNA intermediates. These intermediates concurrently serve as a template for the synthesis of subgenomic (26S) and genomic (49S) RNAs [25,26]. The capsid protein contains two structural domains that interact with RNA in addition to forming the nucleocapsid. The E1 glycoprotein and the E2glycoproteins are arranged in trimeric spikes, (E1-E2)_3_, of heterodimers in *T* = 4 icosahedral symmetry, and the glycoprotein spikes protrude from a host-derived lipid bilayer that surrounds the nucleocapsid core containing the RNAgenome [27]. The glycoproteins play a role in virus entry, egress of mature particles from infected cells, particle stability, and immunogenicity [28,29]. E1 is a class II fusion protein consisting of three structural domains (I, II, and III) that mediate viral membrane fusion via hydrophobic peptides. E2 consists of three structural domains (A, B, and C) that play a key role in binding to cell receptors and are the primary targets of neutralizing antibodies. E3 promotes the folding and activation of p62 (the E2 protein precursor) and stabilizes the spikes formed by E2 and E1. 6K plays roles in virus budding and membrane permeability [27].

## CHIKV genotypes and vectors

As an RNA virus, CHIKV is prone to errors during replication, resulting in a high mutation rate, which may lead to the emergence of novel endemic variants [30]. With this rapid generation of genetic diversity during replication, viral populations consist not just of a single genotype but form mutant clusters of multiple genotypes. Currently, CHIKV is divided into three majorlineages: the West African lineage, the East/Central/South African (ECSA) lineage, and the Asian lineage [31]. Researchers identified CHIKV collected from Indian Ocean region outbreaks (2004–2006) that emerged independently from the ECSA lineage to form the Indian Ocean lineage(ECSA-IOL) [32]. Phylogenetic analysis of ECSA-IOL indicates that it can be further subdivided into four sub-lineages (sl1, sl2, sl3 and sl4) [33].

The geographic distribution of female *Ae. aegypti* and *Ae. albopictus* as CHIKV vectors is strongly associated with the endemic area of CHIKF. *Aedes aegypti* originated in Africa and was later introduced to the New World; it is now distributed mainly in tropical and subtropical areas such as northern Brazil and Southeast Asia [34]. After the 2004–2006 outbreak in the Indian Ocean, it was discovered that *Ae. albopictus* could also serve as a vector for CHIKV [35]. *Aedes albopictus* first appeared in Asia and then spread to islands in the Pacific and Indian Oceans [36]; *Aedes albopictus* can survive in cooler temperatures than *Ae. Aegypti*; therefore, it has a wider geographic distribution and greater risk of transmission [37]; currently, its extent reaches temperate regions such as southern Europe, northern China, southern Brazil, the northern United States, and Japan [38]. In addition, more convenient global transportation has further expanded the geographic distribution of both *Aedes* species and has become a major cause of the global spread of CHIKV.

## Impact of adaptive mutations on vector and population susceptibility to CHIKV

The increased frequency of CHIKV outbreaks and the expansion in its transmission since 2004 have been associated with increased host adaptation due to viral mutations. Studies have shown that adaptive mutations in the natural evolution of CHIKV that alter vector susceptibility are responsible for the dissemination of CHIKV to non-endemic areas as well as large-scale local transmission [39]. The ability to distinguish between these variants will provide a better understanding of the evolutionary tendencies of viruses, explain how viruses develop host jumps [40], and provide a theoretical basis for improving the efficiency of virus control.

### Adaptive changes arising from the natural evolution of CHIKV

#### E1-A226V mutation enhances *Ae. albopictus* susceptibility

A major CHIKF outbreak occurred on Réunion Island in 2005–2006, with 265,000 clinical cases (34% of the population) and 237 deaths reported. As *Ae. aegypti* was virtually absent from the outbreak area but *Ae. albopictus* was abundant and CHIKV RNA was detected in *Ae. albopictus*, it was concluded that *Ae. albopictus* mediated the spread of the outbreak [35]. Characterization of the CHIKV genome revealed a mutation from alanine to valine at E1 glycoprotein 226 (E1-A226V).Infection of *Ae. albopictus* with CHIKV/E1-A226V revealed that the mutant virus enhanced midgut infectivity and viral transmission to the salivary glands and to vertebrates in *Ae. albopictus*, confirming that the E1-A226V mutation resulted in increased viral adaptation in *Ae. albopictus* and gave it a selective advantage over *Ae. aegypti* in accelerating its transmission to uninfected populations [41,42]. This mutation also led to the introduction of CHIKV into new regions, with outbreaks in areas such as Italy and France, where *Ae. aegypti* is absent but *Ae. albopictus* is abundant, and the gradual development of the IOL.

#### A second mutation on a E1-A226V background improves the vector adaptation of CHIKV

In a recent study, researchers used two strains of CHIKV with and without the E1-A226V mutation and found no significant difference in their ability to infect *Ae. albopictus*. This finding suggests that E1-A226V is not the only factor contributing to the enhanced replication of CHIKV in *Ae. albopictus* and that there may be other mutations affecting the vector adaptation of CHIKV [43].

A comparison of CHIKV sequences from outbreaks at the same location (Kerala, India) in 2007 and 2009 by Tsetsarkin *et al.* revealed two new mutations in the background of the E1-A226V mutation, namely E2-K252Q and E2-L210Q, which appeared in Southeast Asia and northeastern India in subsequent years.Researchers infected *Ae. albopictus* with a E2-L210Q mutant strain and found that the mutation increased the infectivity of CHIKV in *Ae. albopictus* midgut cells 2.3–2.4 fold. This mutation represented a stronger effect than that caused by the E1-A226V mutation, which increased the efficiency of CHIKV transmission via *Ae. albopictus* 2 fold, but neither had a significant effect on *Ae. aegypti* adaptation nor led to adaptive changes in primate cells (Vero and 293 cell lines).The mutation did not inhibit virus transmission with *Ae. aegypti* as the vector but increased transmission via *Ae. albopictus*, resulting in increased CHIKV endemicity [44]. Further studies revealed that E2-L210Q and E2-K252Q double mutant viruses spread to the brain of *Ae. albopictus* at higher rates than any of the single mutants, suggesting that the double mutation can lead to further increases in CHIKV host adaptation [33]. However, there is no evidence that the 2009 sub-lineage replaced the 2007 sub-lineage; thus, a more plausible explanation is that the two mutations, E2-K252Q and E2-L210Q, appeared successively and independently in the two outbreaks. Each of these, increased the fitness of *Ae. albopictus* compared to the parental lineage with only the E1-A226V mutation (The structural visualization of mutant residues is shown in Figure 3d) [33].

#### Expression of E1:K211E and E2:V264A on a E1:226A background revealed higher fitness for *Ae. aegypti*

In another study, researchers analysing the sequences of CHIKV isolates endemic to New Delhi, Andhra Pradesh, Tamil Nadu, and West Bengal, India between 2009 and 2010 found two new mutations in the background of E1-226A, namely, E1-K211E and E2-V264A. In *Ae. aegypti*, introduction of single mutation (E1-K211E, E2-V264A) and double mutation (E1-K211E:E2-V264A)in the 226A virus, respectively, resulted in increased viral titres in the midgut, saliva, legs, and wings, all of which were clearly observed after infection. However, in *Ae. albopictus*, introduction of a single or double mutation did not lead to any significant differences in midgut, leg, wing, and saliva titres at either day post infection. The results demonstrated that introduction of E1-K211E or E2-V264A mutations in the background of 226A led to increased infectivity, dissemination, and transmission in *Ae. aegypti* (The structural visualization of mutant residues is shown in Figure 3e) [45,46].

#### nsP4-R82S enhances viral replication in human hosts

An analysis of the CHIKF strain from an outbreak in Malaysia in 2008–2009 by Jolene et al. revealed an arginine-to-serine substitution at amino acid 82 of nsP4 (nsP4-R82S). Phylogenetic analysis indicated a gradual increase in the frequency of this mutation since the start of the 2008 outbreak. The nsP4-R82S sub-lineage was initially present only in Malaysia and Singapore [47], but later it spread to other Southeast Asian countries including Indonesia, Thailand, China, Cambodia, and Myanmar until it replaced the wild-type strain as the main sub-lineage of CHIKV circulating in Southeast Asia in 2009. *Aedes albopictus* is the main vector of CHIKV transmission in Malaysia. Studies in *Ae. albopictus* C6/36 cells have shown that the R82S mutation has no significant effect on viral infection and transmission in *Ae. albopictus* but is more selective in human cell lines. This finding suggests that the mutant strain nsP4-82S causes widespread CHIKF transmission in Southeast Asia by enhancing the fitness of CHIKV in human hosts rather than *Ae. albopictus*, leading to more efficient human-mosquito transmission [48].

#### 3’UTR repeats enhance the replication of CHIKV in mosquitoes

**Figure 2.**
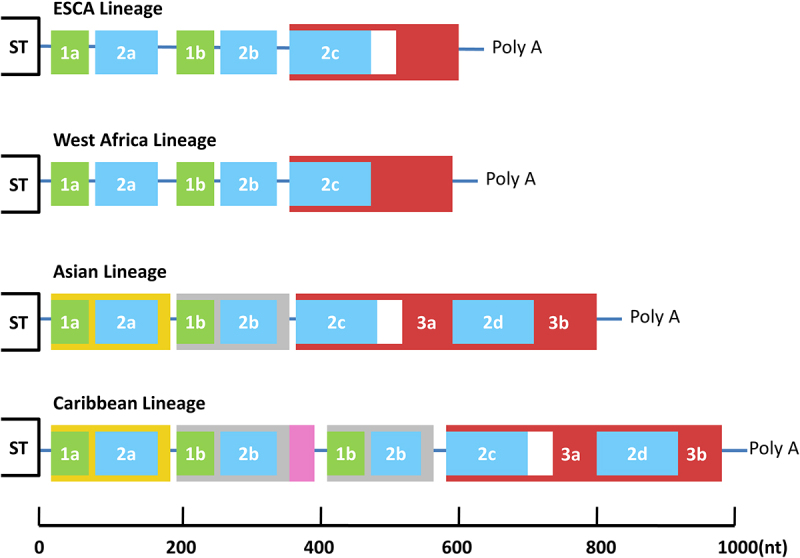
Lineage-specific structures of the CHIKV 3’UTR.

The extensive within-species diversity in the 3’UTR of CHIKV (Figure 2) is unique in this genus of mainly mosquito-borne viruses.In conjunction with studies of the evolutionary trajectory of CHIKV and the relative conservation and comparability of sequences across lineages, this diversity of 3’UTR sequences offers a valuable avenue for elucidating their evolutionary and functional significance. Stapleford *et al.* [49] identified the CHIKV Asian genotype evolved to duplicate a 177 nt repeat element in the 3’UTR of the Caribbean lineage and the Caribbean Lineage 3’UTRreplicates in mammalian cells in a manner similar to another previously reported Asian CHIKV lineage (NC-2011). However, this duplication enhanced replication of the Caribbean lineage in mosquito cells. On this basis, Merwaiss *et al.* [50] used an engineered variant of the Caribbean strain of CHIKV bearing a deletion of the first 500 nt of the 3’UTR to explore the effect of 3’UTR on the virus in mosquitoes. The positive effect of duplications has also been recapitulated by engineering deletion mutants in the backbone of the Caribbean virus. Experimental infection of mosquitoes resulted in delayed replication in the midgut and impaired dissemination. The results of competition assays indicated that the wild-type virus was able to effectively replace the mutant virus during systemic infection. The above results are further confirmed by another study [51]. A series of mutant viruses was engineered based on two wild-type (wt) CHIKV strains, Mal06 (MY002IMR/06/BP; GenBank Acc. No. EU703759.1), representing the Asian lineage, and SL07 (SL-CK1; GenBank Acc. No. HM045801.1), representing the ECSA lineage.The results showed that deletion of any part of the 3’UTR of the Asian Mal06 strain severely reduced its replication rate in mosquito cells. The findings of a study by Claudia et al. [52] also demonstrated a positive correlation between the number of 3’UTR repeats and the rate of viral replication in mosquito cells.Overall, the results of both studies indicate a negative impact of deletion of 3’UTR repeat regions on replication in mosquito cells in vitro.

### Adaptive changes during the experimental propagation of CHIKV

#### E1-V80I:A129V double mutation on a E1-A226V background enhances viral adaptation in *Ae. albopictus*

**Figure 3.**
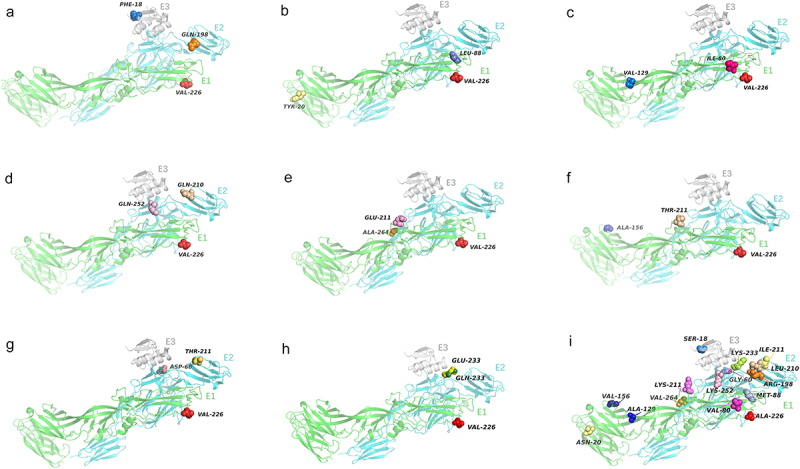
Structural visualization of structural proteins co-mutated residues.

Stapleford *et al.* [53] infected *Ae. aegypti* with a virus containing the E1-A226V mutation isolated from Cambodian patients infected with CHIKV in 2012. Ten days later, the E1-V80I:A129V double mutation was identified in the saliva of all infected mosquitoes. The authors engineered double-mutation V80I:A129V and compared the strains in terms of infection (mosquito midgut), dissemination (legs and wings), and transmission (saliva) in both mosquito hosts. At high-infection doses (10^6^ plaque-forming units [pfu]/ml), infection by and dissemination of the double-mutant virus were significantly higher than those in the control group in *Ae. albopictus*, whereas they were similar to the control group in *Ae. aegypti*. The infectivity,dissemination, and transmission of the double-mutant virus in *Ae. albopictus* were significantly increased at a low infectious dose (10^3^ pfu/ml) compared to controls, but this phenomenon was not observed in *Ae. aegypti*. Animal experiments in eight-day-old mice inoculated with a sublethal dose of the virus (10^2^ pfu) revealed that the double mutation increased the virus titres in blood and muscle 100-fold. Fusion experiments on baby hamster kidney (BHK) cells and mouse embryonic fibroblasts (MEF) showed that the double-mutant virus had significantly higherfusion at the most relevant pH range (pH 5.4–5.8). The E1-A129V single mutant also exhibited a tendency for increased fusion. Incubation of the mutant viruses in cell-free suspensions at 28 °C (mosquito temperature) and 37 °C (mammalian temperature) for 48 h resulted in increased stability at both temperatures of both double-mutant strains. This suggests that the E1-V80I:A129V mutation has a significant adaptive advantage both *in vitro* and *in vivo* in mosquitoes and mammalian hosts, and may be a gene capable of emerging and replacing the parental A226V prevalent strain during natural mosquito-to-mammalian transmission (The structural visualization of mutant residues is shown in Figure 3c) [53].

#### E1-M88L and E1-N20Y mutations on a E1-A226V background enhance viral infectivity

Studies have led to the conclusion that E1-V80 may be a key residue that affects the infectivity and transmission of CHIKV. Further studies found that the mutation E1-V80Q significantly attenuates viral virulence in mammalian and *Ae. aegypti* cells [54]. However, at 14 dpi of *Ae. aegypti* with a low dose (10^4^ PFU/mL) of the mutant virus, virulence was found to be restored in viruses isolated from two mosquitoes. Sequencing revealed that the viruses not only retained the E1-V80Q mutation, but also contained two new mutations (E1-M88L and E1-N20Y). To verify the effects of these two mutations on viral infectivity, ammonium chloride was used to block viral dissemination by neutralizing endosomal and lysosomal pH. The E1-M88L mutant virus was found to infect mammalian BHK-21 cells more rapidly and with greater infectivity, whereas the E1-N20Y mutant virus was not significantly altered. Enhanced infectivity of both E1-M88L and E1-N20Y mutant viruses was observed 60 min after infection of *Ae. albopictus* C6/36 cells. Viral binding assays were performed to confirm whether increased infectivity affects viral binding to cells. The results showed that E1-M88L resulted in increased viral binding compared to the wild-type strain, whereas E1-N20Y had no effect. In addition, compared to E1-N20Y and E1-A226V single mutant viruses, E1-M88L mutant viruses have reduced cholesterol dependence and can enter cells with less cholesterol. In summary, the E1-M88L mutation allows the virus to bind more efficiently to and enter *Aedes* and mammalian cells, which in turn enhances viral infection of *Ae. albopictus* and mammals, whereas the E1-N20Y mutation specifically enhances the infectivity of only *Ae. albopictus* cells, thereby causing increased virulence and enhanced viral transmission (The structural visualization of mutant residues is shown in Figure 3b) [55].

#### E1-V156A and/or K211T mutations on a E1-A226V background enhance viral pathogenicity

To investigate the evolution of CHIKV during transmission from vectors to vertebrate hosts, laboratory *Ae. aegypti* mosquitoes were fed bloodmeal containing the virulent E1-A226V strain, single mosquitoes were isolated at 10 dpi, and the mosquitoes were introduced into cages with suckling mice for 5 days. An additional round of vector-borne transmission was carried out, from infected mouse to naïve mouse via mosquito. Saliva and midgut from all stages of mosquitoes and blood from mice were collected for deep sequencing, which revealed a new V156A mutation in the E1 glycoprotein [53]. These experiments were repeated, and the mutation was stable in both the mosquito and mouse populations [56]. Not only was the E1-V156A mutation detected in two samples collected during a chikungunya outbreak in Rio de Janeiro in 2016 but also was the E1-K211T mutation present in all samples [57].

Membrane fusion experiments in BHK-21 cells revealed that the fusion pH thresholds of the two single mutants V156A and K211T were similar to those of the parental viruses; however, the double mutant E1-V156A:K211T had a lower fusion pH threshold, suggesting that the double-mutant viruses can undergo fusion and enter cells more easily. Previous studies suggested that the E1-K211E mutation increases the adaptability of *Ae. aegypti* [45]. When *Ae. aegypti* was infected with E1-K211T and E1-V156 single-/double-mutant viruses, there were elevated titres of the E1-K211T single-mutant virus in the legs and wings, which may confirm that the mutation of residue 211 is more dominant for infection and facilitates viral transmission in *Ae. aegypti*. Animal experiments in mice infected with single- and double-mutant viruses revealed increased swelling at the site of infection and higher muscle and serum viral titres compared to the parental virus, demonstrating that the E1-V156A and/or K211T mutations resulted in increased viral pathogenicity (The structural visualization of mutant residues is shown in Figure 3f) [56].

#### E2-G60D or E2-I211T on a E1-A226V background enhances ae. Aegypti infectivity

The adaptability of viral populations may not depend only on a single amino acid change; multiple mutations may accumulate during transmission and result in cumulative adaptive effects.Konstantin *et al.* introduced the E1-A226V mutation into three CHIKV strains (Ag41855, LR2006 OPY1 and 37,997) and observed changes in their infectivity. The researchers found that the Ag41855 strain had weaker infectivity of *Ae. albopictus*. Sequence analysis revealed the presence of a unique eight-site mutation in strain Ag41855 compared to the other two strains (Table 1). Preliminary experiments revealed that the Ag41855 strain containing the E2-G60D, E2-I211T and E2-V162A mutations did not show reduced virulence in BHK-21 cells.To further investigate the effects of this mutation on the strain, the E2-V162A mutation was introduced into Ag41855. No significant difference in the infectivity of the virus compared to the parental virus was found. In contrast, the introduction of E2-G60D into Ag41855 resulted in enhanced infectivity of the attenuated strain against *Ae. albopictus*, regardless of whether it was expressed together with E1-A226V. In addition, the E2-G60D mutation alone enhanced the infectivity of CHIKV against *Ae. aegypti* to a level similar to that of the other two strains.When E2-I211T was introduced into an attenuated strain, the mutation was found to enhance the infectivity of the virus against *Ae. albopictus* but had no effect on *Ae. aegypti*.However, this change only occurred when the mutation was co-expressed with the E1-A226V mutation, suggesting that the E2–211 residue acts to specifically regulate the infectivity of CHIKV in *Ae. albopictus*. Phylogenetic analysis showed that the E2-G60D mutation is primarily limited to laboratory strains, but the E2-I211T substitution may be responsible for the emergence of the 1976 South Africa strain, the 2005–2007 Indian Ocean strain, and the 2007 Gabon strain, thus warranting further research (The structural visualization of mutant residues is shown in Figure 3g) [58].

**Table 1.** Genetic differences between Ag41855, LR2006 OPY1, and 37,997 strains of CHIKV.

	Amino acid site	37997	LR2006 OPY1	Ag41855
E1	19	V	V	I
	377	A	A	T
E2	60	D	D	G
	162	A	A	V
	211	T	T	1
nsP3	328	Q	Q	P
	358	S	S	P
	461	P	P	L

#### E2-K233Q/E on a E1-A226V background enhances adaptation of CHIKV in Ae. albopictus

Introduction of E2-K233Q in the background of E1-226 V increased the efficiency of CHIKV infection of *Ae. albopictus* 5 fold. Further testing of the effect of the E2-K233Q mutation on viral infection and replication in the midgut of *Ae. albopictus* revealed that the relative amount of mutant virus RNA in the midgut was increased 3.6 fold at 1 dpi, progressively increasing to 8.9 fold at 3 dpi, indicating that the E2-K233Q mutation plays a role in the initial stages of infection and/or replication in the midgut [33]. In the study by Tsetsarkin *et al.*, the GenBank database was searched for the E2-233Q mutation, and although it was not found, a mutant sequence with E2-233E was found instead. The introduction of E2-K233E into the E1-226 V background resulted in a 5.7-fold increase in the relative amount of viral RNA in the midgut at 1 dpi. Mosquitoes that ingested bloodmeal containing the double-mutant virus exhibited disseminated viral infection not only in the head but also in the mosquito homogenate, suggesting that E2-K233E increases the adaptation of CHIKV in other organs/tissues of the mosquito in addition to its effects on midgut infection (The structural visualization of mutant residues is shown in Figure 3h) [33].

#### Two double mutations on a E1-A226V background enhance adaptation of CHIKV in Ae. albopictus

The double mutation that emerged in the ECSA-IOL sl1 sub-lineage (nsP2-L539S/E2-K252Q) was introduced into the E1-226 V mutant strain. Measurements of viral infection in mosquito heads showed that the experimental viruses were significantly more efficient at disseminating infections via *Ae. albopictus* compared to the control group, in which only the E1-A226V mutation was present. In addition, the double-mutant virus resulted in a 7.8-fold average RNA increase in the *Ae. albopictus* midgut 10 days post infection (dpi). To further validate these results, the sl1 virus strain Thailand-2009_CK11/53 containing the characteristic nsP2-L539S/E2-K252Q substitution isolated from febrile patients was analysed. The results showed that viruses containing the double mutation had 5.8-fold greater transmission efficiency and that the *Ae. albopictus* viral titres at 10 dpi were higher than that of those control group. These results confirm that nsP2-L539S/E2-K252Q increases viral adaptation in *Ae. albopictus* infection. Introduction of a non-synonymous mutation in sl3 (E2-R198Q/E3-S18F) into E1-226 V increased the transmission ability of the experimental group of *Ae. albopictus*11.5 fold compared to the E1-226 V control. In addition, the relative amount of RNA in the midgut of mosquitoes infected with the double-mutant virus was increased 10.2-fold compared to the control group. These results indicate that the sl3 strain achieved further adaptation in *Ae. albopictus* after acquiring the two synergistic mutations E3-S18F and E2-R198Q (The structural visualization of mutant residues is shown in Figure 3a) [33].

#### Deletion of the 3’ UTR and E2-K200R enhances CHIKV virulence and transmission

CHIKV was isolated from the serum of mice persistently infected with CHIKV for 28 days and then inoculated into the left hindfoot pad of wild-type mice. These mice developed signs of more severe musculoskeletal lesions, suggesting that this virus is more virulent than its parent virus. Sequencing of this virus revealed three mutations: a substitution at residue 200 of the E2 protein (E2-K200R), a synonymous 10506A→G mutation in the E1 gene, and a 44-nt (11,921 –11,964) deletion in the 3’ UTR (Δ3’UTR). The E2-K200R mutation was also found in a Thai case in 1995, whereas the 44-nt deletion is highly conserved and has only been observed in the Asian lineage [59].

To confirm that these mutations were responsible for increased virulence, a E2-K200R:Δ3’ UTR double mutant virus was constructed and inoculated into the footpad of mice. The increased degree of weight loss and more severe musculoskeletal symptoms in mice infected with the double-mutant virus compared to controls confirmed that the mutations were responsible for the damage to musculoskeletal tissues distant from the inoculation site and aggravated systemic disease. Next, the transmissibility of the mutated virus was assessed, and the mutation was found to enhance the early transmission of CHIKV from the inoculation site to other sites. *In-vitro* experiments revealed that the amount of the virus detected in the experimental and control groups was similar in mouse fibroblasts, differentiated C2C12 mouse cells, and C6/36 mosquito cells, suggesting that the mutation had negligible effect on viral replication and mainly affected viral transmission in mice. Viruses with the E2-K200R or Δ3’UTR mutations were constructed, and it was found that the E2-K200R single mutation also increased viral transmissibility and pathogenicity, but the 44-nt deletion in the 3’ UTR had to be present in conjunction with the E2-K200R mutation in order to enhance viral pathogenicity [59].

#### Non-structural protein mutations on a E1-A226V background enhance viral adaptation in *Ae. albopictus* and vertebrates

CHIKV was sequenced after 19 blind passages in difluoromethylornithine (DFMO)-treated cells, and three mutations were identified, with two in nsP1 (G230R and V326M) and one in which the stop codon between nsP3 and nsP4 was mutated to arginine (*524 R). Mutant viruses were constructed and used to infect Vero-E6 cells, after which plaque formation experiments were performed. Viruses with the nsP1 mutation resulted in smaller plaques, and viruses with the nsP1/*524 R triple mutation resulted in larger plaques. Viral infection experiments in *Ae. albopictus* C6/36 cells revealed that each of the single mutant viruses (G230R, V326M, and *524 R) exhibited severe defects in replication, whereas the viral titres of the triple mutants were similar to wild-type levels. To investigate their adaptation, each single-mutant virus was mixed with wild-type CHIKV at a 1:1 ratio and used to infect BHK-21 or C6/36 cells for 24 h. Sequencing of the target mutations after five passages revealed that the single mutant was lost and that wild-type and triple-mutant viruses with the *524 R mutation were present in BHK-21 cells, suggesting that the single-mutant virus was more poorly adapted compared to the wild-type virus, whereas the viruses with the *524 R mutation were better adapted. After observing that the mutant viruses exhibited an adaptive advantage *in vitro*, *Ae. albopictus* mosquitoes and zebrafish, a vertebrate infection model of CHIKV, were infected with mutant CHIKV. The results showed that single-mutant viruses had decreased titres in mosquitoes compared to the wild-type, whereas the titres of the triple-mutant virus were significantly higher. These results indicate that single-mutant CHIKV exhibits growth defects in mosquitoes, but that triple-mutant viruses have enhanced replication and adaptation in mammals and *Ae. albopictus* both *in vitro* and *in vivo* [60].

The nsP1:R171Q mutation on a background E1-A226V was identified in virus isolates from CHIKF outbreaks in Comoros in 2005, in Sri Lanka in 2006, in India in 2008–2013, and in the Caribbean in 2014. Although the function of this mutation has not been elucidated, there is already evidence that the nsP1:171Q mutant exhibits enhanced pathogenicity in primate cells [61].

### Other potentially adaptive mutations arising through evolution

Partial sequencing of the E1 region of CHIKV during the outbreak in the southern Indian state of Karnataka between November 2015 and November 2016 and alignment with other ECSA strains from India revealed six non-synonymous mutations: K211E, M269V, D284E, V322A, I317V, and V220I [62]. The K211E mutation facilitates enhanced host adaptation, as discussed earlier. The M269V and D284E mutations, which were reported in the 2005–2006 outbreaks in India, have been retained in currently prevalent strains, suggesting that they may be associated with host adaptation, but their specific effects have not been experimentally confirmed. The I317V mutation, which was previously found only in north-central India and Bangladesh, was first continuously transmitted in southern India, and deletion of the I317V mutation was observed in the 2020–2021 outbreak in Pune, India. Whether the I317V mutation affects CHIKV transmission is unknown, but absence of the I317V mutation may limit CHIKV transmission and may be a reason for why the number of cases in the 2020–2021 outbreak in Pune, India was lower than the number of cases in 2016 [63].

Comparing the sequences obtained from cases during the 2014–2016 CHIKF outbreak in Mexico with those sampled at the beginning of the outbreak, 14, 7, and 4 non-synonymous mutations were found in nsP3, E2, and E1, respectively, compared to that in the ECSA genotype, and four and one non-synonymous mutations were found in nsP3 and E2, respectively, compared to that in the Asian genotype. Three of these non-synonymous mutations (E2-S248F, nsP3-A437T, and nsP3-L451F) were also found in outbreaks in the Caribbean and the Philippines in 2012. It has been demonstrated that E2-S248L favours viral transmission in *Ae. aegypti*, whereas further studies are needed to determine whether the E2-S248F mutation affects host adaptation. The nsP3-A437T and nsP3-L451F mutations have not been studied in detail; however, the presence of the A437V mutation, at the same locus as nsP3-A437T, has been reported in previous ECSA samples, suggesting that the amino acid change at this locus may be similar to that of the E2-S248F mutation discussed earlier and affect the adaptation of *Ae. aegypti* and *Ae. albopictus* [64].

## Concluding remarks

As an RNA virus, the adaptive mutations that occur during CHIKV replication impact the susceptibility of vectors and humans and are a major factor in the increasing global spread of CHIKF (Table 2). To date, the ECSA genotype has dominated, been widely prevalent, and evolving globally [39]. The 2004–2006 outbreak of CHIKF in the Indian Ocean region was identified as being caused by a newly emerged branch of the ECSA lineage, known as ECSA-IOL [65]. ECSA-IOL is prominently characterized by the presence of the E1-A226V mutation, which confers a selective advantage to the virus in *Ae. albopictus* [40], resulting in the increasing global spread of IOL sub-lineages and leading to over six million infections in subsequent years [35].

**Table 2.** Chikungunya fever outbreaks and viral gene mutations.

Genotype	Year of outbreak	Outbreak area	Main vector	Mutation
ECSA	1953	Tanzania [66]	*Ae. aegypti*	
	1976	South Africa [66]	*Ae. aegypti*	
	1982	Central Africa, Uganda[66]	*Ae. aegypti*	
	2000	India [67]	*Ae. aegypti*	**nsP1**:V326M, L507R
				**nsP2**:H374Y
				**nsP3**:Q328P, S358P, I376T, C435R, V438A, M449T, S462N
				**nsP4**:Q500L
				**E1**:V19I, M269V, A377T
				**E2**:A162V, A164T, V318M, I377V
				**E3**:I23T
				**C**:K63R
	2002	Equatorial Guinea [68]	*Ae. aegypti*	
	2004	Kenyan [7]	*Ae. aegypti*	
	2005	La Réunion [69]	*Ae. albopictus*	**nsP1**: T301I
				**nsP2**:Y642N
				**nsP3**:S358P, 460del
				**E1**:A226V
				**E2**:Q146R
	2006	India [67]	*Ae. albopictus*	**nsP1**:A101V, T128K, T376M, T376M, Q488R, L507R
				**nsP2**:S54N, H374Y, T675M, A793V
				**nsP3**:V175I, Y217H, V331A, T337I, T341M, I376T, L461P, P471S
				**nsP4**:T75A, T254A, Q500L
				**E1**:K211N, V213I, M269V, D284E
				**E2**:A164T, I211T, T312M, S375T, V386A
				**E3**:I23T
				**6K**:V8I
				**C**:P23S, V27I, K63R, N80D
	2007, 2017	Italy [70,71]	*Ae. albopictus*	**E1**:A226V
	2007	Gabon [72]	*Ae. albopictus*	**E1**:A226V
	2008	China[73]	*Ae. albopictus*	**nsP1**:Q120R
				**nsP2**:G577R, N632S, L539S
				**nsP3**:M394I, D372N,
				**nsP4**:P181S
				**E1**:A306V, A226V
				**E2**:R178H, K252Q
				**6K**:V31I
				**C**:T8A
	2008, 2013, 2018	Thailand [74]	*Ae. albopictus*	**E1**:K211E
				**E2**:V264A
	2008	Malaysia [75]	*Ae. albopictus*	**nsP1**:S3P, S34P, T128K, V153I, M253K, T376M, G454S, R473S, A478T, N486D, Q488R, Q491R, H507R
				**nsP2**:L16P, S54N, S218T, L273Q, M338K, H374Y, V466M, V486I, L539S, I756V, S768N, A793V
				**nsP3**:T77S, G117R, V175I, I176V, V213M, N283S, V303T, V331A, R332Q, V334A, M336T, T337I, A349V, T353I, del376-382THTLPST, I383T, I413T, Q434L, A437V, I449M, R452Q, I457T, T458A, V459T, L461P, S462N, P471S, D483N, D484E
				**nsP4**:L42A, T58M, T75A, K85R, A90S, V101T, R235Q, T254A, R271K, D280E, A366T, Q500L, A582V
				**C**:P23S, V27I, K37Q, V48A, K73R, R78Q, M81T, V93A
				**E1**:S72N, T98A, A145T, E211K, S225A, A226V, M269V, D284E, S304P, P397L
				**E2**:I2T, H5N, G118S, R149K, A157V, A164T, S194G, D205G, S207N, S248L, K252Q, V/L255I, T312M, I317V, R318V, S375T, V384M, V386A
				**E3**:K33E, S44R, R60H, R62Q
				**6K**:V8I, M45T, T47A, L52M
	2009	India [44]	*Ae. albopictus*	**E2**:L210Q, K252Q
	2010, 2014	France [76]	*Ae. albopictus*	
	2011	Cambodia[77]	*Ae. albopictus*	
	2011	Republic of Congo [78]	*Ae. aegypti,*	**E1**:A226V
			*Ae. albopictus*	
	2011	Indonesia [79]	*Ae. albopictus*	**E1**:A226V
	2012	Bhutan [80]	*Ae. aegypti,*	
			*Ae. albopictus*	
	2016	Pakistan[80]	*Ae. albopictus*	**nsP1**:K128T
				**E1**: T98A, S111T, A145T, K157N
	2016	Brazil	*Ae. aegypti*	**E1**: V156A, K211T
	2017	China [81]	*Ae. albopictus*	**nsP3**:D372E
				**E1**:M269V, D284E
WA	1964	Nigeria [82]	*Ae. aegypti*	
	1966, 1983	Senegal [82]	*Ae. aegypti*	
Asian	1958, 1988, 1995, 1990	Thailand [66]	*Ae. aegypti*	
	1963, 1973	India[67]	*Ae. aegypti*	
	1983,2008, 2011	Indonesia [79]	*Ae. albopictus,Ae. aegypti*	
	1998, 2006	Malaysia[75]	*Ae. albopictus,Ae. aegypti*	
	2011, 2013	New Caledonia [83]	*Ae. aegypti*	
	2014	Tonga, American Samoa, the Independent States of Samoa, Tokelau [83]	*Ae. aegypti*	
	2014	French Polynesia [84]	*Ae. aegypti,*	
			*Ae. polynesiensis*	

To address the threat to human health posed by CHIKF, attention should be paid to host-associated adaptive changes in the virus. Although the mechanisms of adaptive evolution of viruses have not been fully understood, many studies have demonstrated the close relationship between viral mutations and changes in host adaptation, and new genotypes of mutant strains may cause large-scale outbreaks of CHIKF. For example, the E1-A226V and E2-L210Q mutations that appeared during natural evolution enhance the infectivity of *Ae. albopictus* [41,44]. The amino acid sites in which structural proteins undergo synergistic mutations are shown in the Figure 3i. Of these, E1-A226V is widely believed to have led to the expansion of CHIKV transmission. Subsequently, several secondary mutations have been identified (A226V:E2-L210Q, A226V:E2-K252Q, nsP2:L539S/E2-K252Q, and E2-R198Q/E3-S18F) that further enhance viral adaptation in *Ae. albopictus* [33], whereas E1-V156A, E1-K211T, E1-K211E and E2-V264A enhance adaptation in *Ae. aegypti* [45,56], and nsP4:R82S increases the efficiency of human-mosquito transmission. Various adaptive mutations have also been identified during laboratory passage. E2-G60D, E2-I211T, E1-V80I:A129V, and E2-K233Q/E, which appear in the background of the E1-A226V mutation and further enhance viral adaptation in *Ae. albopictus* [53,58]; E2-K200R:Δ3’UTR enhances viral adaptation, virulence, and transmission in mammals [59]. The nsP1:G230R/V326M/nsP3:*524 R triple mutant has enhanced viral replication and adaptation in mammals and *Ae. albopictusin vitro* and *in vivo* [60](Table 3). The fact that secondary mutations can further enhance viral adaptation suggests that adaptive changes to CHIKV may also be found in *Ae. albopictus*. However, it is worth noting that many ECSA isolates from recent outbreaks do not carry the E1 A226V mutation. In addition to the 3’UTR repeats present in the Caribbean strains mentioned above, Souza et al. sequenced CHIKV endemic to Tocantins, Brazil, between 2021 and 2022 and found that all 27 sequences belonged to the ECSA lineage, with 11 identified highly frequent missense mutations, while none of them had the E1-A226V substitution described earlier [85].

**Table 3.** Amino acid mutations enhance host adaptation by CHIKV.

Gene	Mutation	Genotype	Effect
E1	A226V	IOL	Increase adaptation and infectivity to *Ae. albopictus* [41]
	K211E	IOL	Increase adaptation to *Ae. aegypti* [45]
	V80I/A129V	IOL	Increase fitness for *Ae. albopictus* on a E1-A226V background [53]
	V80Q/M88L	IOL	Increase adaptation and transmission to *Ae. aegypti* and mammals on a E1-A226V background [55]
	V80Q/N20Y	IOL	Increase adaptation and transmission to *Ae. aegypti* on a E1-A226V background [19]
	V156A	ESCA (nature)	Increase pathogenicity of the virus by *Aedes aegypti* on a E1-A226V background [56]
	K211T	IOL (experiment)	
E2	G60D	ESCA	Increase fitness in Ae. albopictus and *Aedes aegypti* on a E1-A226V background [58]
	R198Q	IOL	Enhanced infection of Ae. albopictus, synergistic with E3:18F on a E1-A226V background [33]
	K200R	Asian	Enhanced viral transmissibility and pathogenicity in mammals [59]
	L210Q	IOL	Enhanced infection of *Ae. albopictus* on a E1-A226V background [44]
	I211T	IOL	Increase fitness in Ae. albopictus on a E1-A226V background [58]
	K233E/Q	IOL	Enhanced infection of Ae. albopictus on a E1-A226V background [33]
	K252Q	IOL	Enhanced infection of *Ae. albopictus*, synergistic with nsP2: L539S on a E1-A226V background [33]
	V264A	IOL	Enhanced fitness in Ae. aegypti on a E1-226A background [45]
E3	S18F	IOL	Enhanced infection of *Ae. albopictus*, synergistic with E2:R198Q on a E1-A226V background [33]
nsP1	G230R	IOL	Increase replication in Ae. albopictus in combination with nsP3:524* on a E1-A226V background [60]
	V326M		
nsP2	L539S	IOL	Enhanced infection of Ae. albopictus, synergistic with E2-K252Q on a E1-A226V background [33]
nsP3	*524 R	IOL	Enhanced infection of Ae. albopictus, synergistic with nsP1: G230R/V326M on a E1-A226V background [60]
nsP4	R82S	ESCA	Increase human-mosquito transmission [48]
3’UTR	Deletion (11921 –11,964 nt)	Asian	Increase viral pathogenicity for mammals compared to the E2-K200R single mutation [59]

With the frequent movement of mosquitoes between endemic and non-endemic areas worldwide, it is likely that variants with greater infectivity and transmissivity will emerge in the future. Therefore, improved monitoring of genomic variations in CHIKV and focusing on mutations occurring during its evolution are important for controlling the global spread of CHIKV. Equally valuable is the introduction of adaptive mutations of CHIKV in the laboratory. Identifying new viral subtypes in nature can take years, while the function of newly discovered mutation sites can be validated in the laboratory through cell infection and *in vivo* mosquito-mouse transmission experiments. This approach would facilitate the targeted surveillance of CHIKV evolution by health agencies such as the CDC, enabling good responses to CHIKF outbreaks that can mitigate the impact of CHIKV mutations on public health.

## Data Availability

No primary data is included in this article.
